# Cre-mediated cellular modification for establishing producer CHO cells of recombinant scFv-Fc

**DOI:** 10.1186/1753-6561-9-S9-P5

**Published:** 2015-12-14

**Authors:** Yoshinori Kawabe, Takanori Inao, Shodai Komatsu, Akira Ito, Masamichi Kamihira

**Affiliations:** 1 Department of Chemical Engineering, Faculty of Engineering, Kyushu University, Fukuoka, 819-0395, Japan; 2 Graduate School of Systems Life Sciences, Kyushu University, Fukuoka, 819-0395, Japan

## Background

The targeted integration of transgenes into a pre-characterized genomic locus enables predictable protein expression to occur, which reduces the need for the screening of transfected clones. We previously developed an accumulative site-specific gene integration system (AGIS), which enabled the repeated integration of multiple transgenes into a pre-determined locus of the cell genome [1,2]. We achieved the repeated integration of recombinant scFv-Fc gene into the genome of Chinese hamster ovary (CHO) cells, a common animal host cell for the production of recombinant biopharmaceutical proteins. Productivity was shown to correspond to the copy number of the expression cassette [3]. Cell screening after gene transfection was the most time-consuming process in AGIS, but we hypothesized that the use of fluorescent selection markers instead of drug resistant genes would facilitate the cell establishment process because Cre-mediated integration is completed within 48 h post-transfection. Therefore, the present study used marker genes encoding fluorescent proteins to speed up the establishment of producer CHO cells using AGIS.

## Materials and methods

We constructed a donor plasmid encoding a DsRed gene upstream of a scFv-Fc expression unit flanked by a wild-type loxP and a mutated loxP (P2R-scFvFc). An insulator element derived from the CHO cell genome was located either side of the scFv-Fc expression cassette. Recombinant CHO cells (CHO/P1G) containing EGFP flanked by compatible target sites were used as founder cells [4]. CHO/P1G cells were seeded at 1.2 × 105 cells per well of 24-well plates, and the donor plasmid and a Cre expression vector (pCEP4/NCre) were co-transfected into the founder cells the next day using Lipofectamine 2000 (Life Technologies) according to the manufacturer's instructions. After 48 h, transfected cells were seeded at 2,400 cells per 100-mm dish. Colonies exhibiting a shift in fluorescence from green to red were identified by fluorescence-activated cell sorting (FACS) to establish cell clones. Transgene integration into the cells was confirmed by genomic PCR analysis. The viable cell density was determined by the trypan blue exclusion method. Recombinant scFv-Fc concentration was measured by ELISA.

## Results and discussion

**Figure 1:**
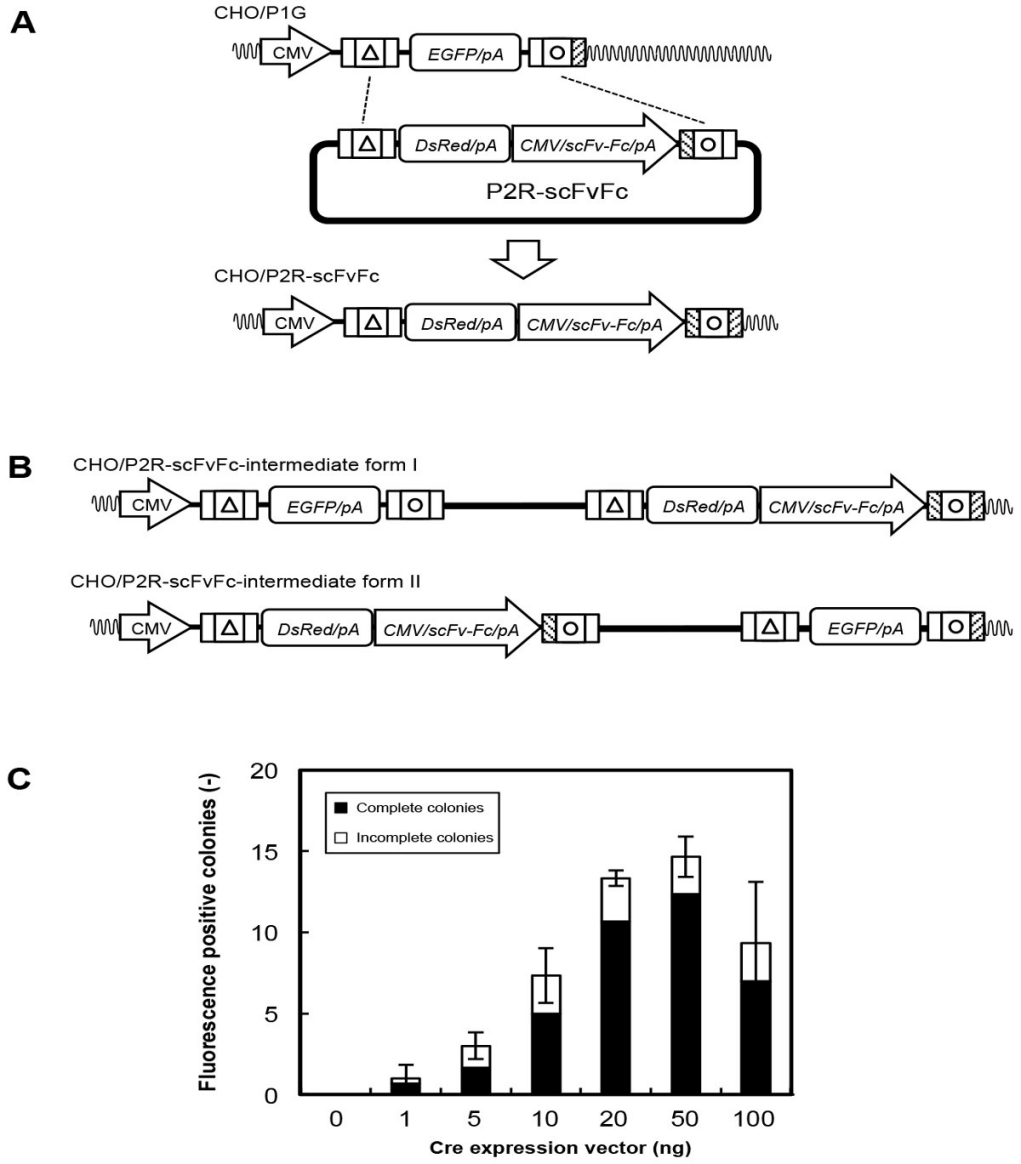
**Targeted transgene integration for CHO cells using Cre/*loxP***. **A: **Schematic of Cre-RMCE in CHO/P1G cells. **B: **Intermediate clonal forms during Cre-RMCE. **C: **Colony numbers undergoing a shift in fluorescence

Following transfection of the donor plasmid with a Cre expression vector (5 ng) into founder cells, we screened recombinant cells exhibiting a fluorescent color shift from green to red via the Cre/loxP reaction (Figure 1A). A total of 44.4% of colonies exhibited an incomplete fluorescence shift, with some DsRed/EGFP double positive cells or weakly DsRed-positive cells observed. A two-step recombination reaction took place, involving the appearance of intermediate clones in a Cre-recombinase mediated cassette exchange (RMCE) reaction, as shown in Figure 1B. Thus, an increased amount of the Cre expression vector increased the number of DsRed-positive colonies, reducing the population of incomplete colonies. When 50 ng of Cre expression vector was used, the maximum integration efficiency for complete clones was observed (84.1%) (Figure 1C). The integration efficiency decreased when >50 ng Cre expression vector was used because of its cytotoxicity. For established clones (CHO/P2R-scFvFc), transgene integration into the expected chromosomal site was confirmed by genomic PCR and sequencing of amplicons. No significant difference in cell growth was observed among the clones. They also exhibited similar levels of scFv-Fc productivity and a similar fluorescence profile. Cells harboring scFv-Fc expression units flanked by insulator elements showed higher and more stable scFv-Fc productivity compared with those without the insulator insertion.

## Conclusions

Transgenes were integrated into a predetermined chromosomal locus of CHO cells using AGIS. The rapid screening of recombinant cells using a FACS device was based on a shift in fluorescence. RMCE-completed clones exhibited similar levels of scFv-Fc productivity and fluorescent protein expression. Moreover, higher and more stable scFv-Fc production was achieved using the insulator insertion.

## Acknowledgements

This work was partially funded by a project to build infrastructure for creating next-generation drugs for personalized medicine from the Ministry of Economy, Trade and Industry (METI), Japan; and the Kato Memorial Bioscience Foundation. The insulator element derived from the CHO cell genome was provided by Toyobo (Tsuruga, Japan).
